# Medical Application of* Spirulina platensis* Derived C-Phycocyanin

**DOI:** 10.1155/2016/7803846

**Published:** 2016-05-11

**Authors:** Qian Liu, Yinghong Huang, Ronghua Zhang, Tiange Cai, Yu Cai

**Affiliations:** ^1^College of Pharmacy, Jinan University, Guangzhou 510632, China; ^2^College of Life Sciences, Liaoning University, Shenyang 110036, China; ^3^Cancer Institute of Jinan University, Guangzhou 510632, China

## Abstract

Along with the development of marine biological pharmaceutical research, high-effective and low-toxic drugs and functional foods isolated from marine organisms have become a new field of pharmacy and bromatology. The pharmacological actions, such as anti-inflammation, antioxidation, antitumor, immunological enhancement, and hepatorenal protection of C-phycocyanin (C-PC) from* Spirulina platensis*, have been reported, and C-PC has important value of development and utilization either as drug or as functional food. There are many researches about the various pharmacological actions and mechanisms of C-PC, but related reports are only to some extent integrated deeply and accurately enough, which put some limitations to the further application of C-PC in medicine. Particularly, with the improvement of living standards and attention to health issues, C-PC being a functional food is preferred by more and more people. C-PC is easy to get, safe, and nontoxic; thus, it has a great potential of research and development as a drug or functional food. Here, the separation and purification, physicochemical properties, physiological and pharmacological activities, safety, and some applications are reviewed to provide relevant basis for the development of natural medicine and applied products.

## 1. Introduction

Phycocyanin (PC) is a light-harvesting, pigment-binding protein isolated from algae [1]. PCs isolated from blue-green algae and red algae are classified as C-PC and R-phycocyanin (R-PC), respectively [2, 3]. C-PC exists in many species of blue-green algae, red algae, Cryptophyta, and few dinoflagellates. Among these sources of C-PC,* S. platensis*,* Anabaena*, and other blue-green algae display high C-PC contents.

**Figure 1:**
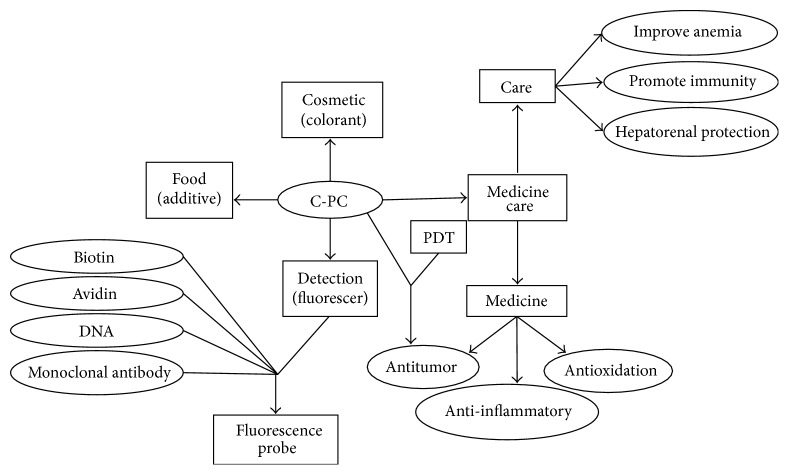
Application of C-PC.

C-PC obtained from* S. platensis* has been widely used as a food additive and cosmetic colorant in Japan [4]. C-PC has also been utilized as a medicine [5, 6], food [7], colorant [8], and fluorescent dye [9]; some of the major studies on C-PC application are illustrated in Figure 1. Among these applications, the use of C-PC in medicine and biology has attracted increasing attention. First, studies have demonstrated that C-PC functions in antioxidation [9], inflammation [10], antitumor [11], and immunity enhancement [12]. In addition, C-PC can be processed into a fluorescent reagent, fluorescent probe, and fluorescent tracer, which are used in medical diagnosis, immunology, biological engineering, and other research fields because of its intense fluorescence [13, 14]. C-PC is also a nontoxic photosensitizer that can be used in adjuvant therapy in the photodynamic therapy (PDT) of tumors [15].

Considering the good development prospect and high content of C-PC (up to 10–20%) in* S. platensis*, we review studies on the isolation and purification, physicochemical properties, physiological and pharmacological activities, safety, and other aspects of* S. platensis* to provide a relevant basis for the development of natural medicine and applied products.

## 2. Isolation and Purification of C-PC

### 2.1. Isolation

C-PC is water-soluble and insoluble in alcohol and esters. Thus, the water extraction method was adopted. Some popular approaches include the following. (a) In repeated freezing and thawing method [16],* S. platensis* powder is dissolved in water at a certain proportion, quickly frozen at −20°C, and then thawed at 5°C thrice; the cell breaking rate can reach up to more than 90%. This method is simple, but time-consuming and wasteful in powder when processing a large number of samples. (b) In direct osmosis [17],* S. platensis* powder is immersed in distilled water, low-salt-concentration solution, or buffer solution for a night, causing* S. platensis* cell wall to break automatically; this method is simple but time-consuming. (c) In the ultrasonic method [18, 19],* S. platensis* solution is treated with ultrasound to accelerate cell wall breaking through direct osmosis. This method effectively shortens the treatment time. (d) In the chemical reagent method [20], some chemical reagents, such as anionic surface active agent, are utilized to destroy the cell membrane, and protein is dissolved under mild conditions. In this method, the cell wall remains intact and the purity of C-PC is improved. (e) In enzyme digestion [21], potassium chloride lysozyme is used to break* S. platensis* cell wall, improving the extraction yield. This method only needs a short time and is a suitable method to process a large number of samples, but it has strict requirements for temperature, pH, and other experimental conditions. Aqueous two-phase extraction has been recently developed, and its application in the separation of protein, polysaccharide, nucleic acid, DNA, and other molecules has increasingly become common [22–24]. Chethana et al. [25] achieved an extraction rate of 79% and purity of 4.32 by using single step aqueous two-phase extraction. This approach shortens the processing time, lowers the processing cost, achieves a stable biological activity, and can be directly extended up to industrial scale; thus, this method offers great application prospects. Moreover, extraction by reverse micelle offers the advantages of high selectivity, high material activity, and coinstantaneous separation and concentration of raw material [26]. Liu et al. [27] processed the aqueous extract of* S. platensis* (pH 7.0 with 0.1 mol/L KCl) by using cetyltrimethylammonium bromide (CTAB)/1-amyl alcohol-1-octane (at a volume ratio of 4 : 1) and found that reverse micelle demonstrates a C-PC extraction rate of 96.3%.

### 2.2. Purification

The C-PC crude aqueous extract mentioned above contains many other water-soluble components, such as* S. platensis* polysaccharide, water-soluble vitamins, and proteins; the purity of proteins determined at an absorbance ratio of 620 and 280 nm (*A*620/*A*280) is above 2.0 and 4.0, respectively, indicating that C-PC can be applied in the food industry and medical field [28]. Therefore, further purification of C-PC crude aqueous extract is necessary. The following are some of the general purification methods. (a) In the salting out method, different proteins are separated step by step in accordance with the principle of separation of different substances from varying concentrations of salt solution [29]. Patel et al. [30] employed a two-step precipitation method using 25–50% saturated ammonium sulfate to obtain C-PC from* S. platensis* water extract. (b) Isoelectric point precipitation is used to separate target proteins and other proteins by adjusting the pH of a solution to the isoelectric point of a protein on the basis of the different isoelectric points of various proteins. (c) Chromatographic methods, such as hydroxylapatite (HA) chromatography [31, 32], Sephadex chromatography [33], and ion exchange chromatography [34], are currently preferred for purification. Wang et al. [35] achieved C-PC purity of 14.47 by using Sephadex G-200, DEAE-Sephadex A-25, and HA.

## 3. Physicochemical Properties of C-PC

### 3.1. Spectral Property

Spectral property is a significant property of C-PC being a light-harvesting pigment protein and is used as a basis for its simple and effective identification and quality control.  Table 1 shows the main optical properties [2]; among these properties, ultraviolet absorption was determined for the identification and purity of C-PC. Yu et al. [36] determined the maximum absorption peak and fluorescence emission peak at 625 and 648 nm, respectively, which are close to the data listed in Table 1. Yin et al. [2, 37] obtained the infrared spectra of C-PC and detected the absorption peaks at 1650, 1550, 1100, 1050, 3200, and 650 cm^−1^, which provide further basis for the C-PC identification.

### 3.2. Structure and Amino Acid Composition

The study of amino acid composition of C-PC can pave the way for further exploring the inner structure and active groups and also provide a theoretical basis for other properties. Yin et al. [2, 37], Liu et al. [38], and Li et al. [14] determined the amino acid composition of C-PC obtained from* S. platensis*, and their results revealed that the amino acid composition of different strains in C-PC is basically the same. C-PC includes *α* and *β* subunits which are composed of 162 and 172 amino acids, respectively [39]. The following shows amino acid sequence of *α* and *β* subunits:

  
*α* chain of C-PC isolated from* Spirulina platensis*:
  mktplteavsiadsqgrflssteiqvafgrfrqakagleaakaltskadslisgaaqavynkfpyttqmpGpnyaadqrgkdkcardigyylrmvty cliaggtgpmdeyliagideinrtfelspswyiealkyikanhGlsgdaateansydyainals
  
*β* chain of C-PC isolated from* Spirulina platensis*:
  mfdaftkvvsqadtrgemlstaqidalsqmvaesnkrldavnritsnastivsnaarslfaeqpqliapgGnaytsrrmaaclrdmeiilvyvtyavf agdasvledrclnglretylalgtpgssvavgvgkmkeaalaivndpagitpgdcsalaseiasyfdracaavs

### 3.3. *α* and *β* Subunits of C-PC

The present study indicated that C-PC mainly consists of *α* and *β* subunits forming the polymer (*αβ*)_*n*_ (*n* = 1–6) [40], which then combines with the blue algae-derived pigment. The molecular weight of these subunits varies. The purified C-PC was analyzed through 12% sodium dodecyl sulfate polyacrylamide gel electrophoresis, and the results revealed that* S. platensis* derived C-PC is composed of *α* and *β* subunits with molecular weights of 14500 and 15000 *μ*, respectively [41]. However, Yu et al. [36] reported different results, in which the molecular weights of *α* and *β* subunits are 14900 and 17200 *μ*, respectively. Peng et al. [42] determined the relative migration rate (*x*) of standard protein and the corresponding molecular weight of log⁡(*y*) for regression analysis, and the regression equation is *y* = 1.0228*x* + 5.1255 (*R* ^2^ = 0.9889). The molecular weights of *α* and *β* subunits were about 16.3 and 18.9 kDa, respectively, consistent with previous results [43, 44].

### 3.4. Stability of C-PC

 Li et al. [21] reported that C-PC is stable under 40°C; at temperatures above 40°C, the pigment begins to decompose and the optical density decreases gradually, whereas the optical density drops sharply at temperatures above 50°C and optical density reduced by 75% at temperatures above 70°C. Furthermore, they found that sugar solution can improve the stability of C-PC under heat. Light slightly affects the C-PC and the optical density of C-PC solution does not change under 5000 1x for 60 h and pH 5. The study also revealed that the color and optical density are constant between pH 4.0 and pH 8.5, whereas the color of C-PC solution begins to fade when pH is higher than 8.5 or lower than 4. All of the results above indicate that C-PC is sensitive to temperature and pH but not to light. This finding is essential to control the conditions for C-PC extraction and purification.

## 4. Application of C-PC in Tumor

### 4.1. Antitumor Mechanism of C-PC

#### 4.1.1. Influence on Cell Cycle

**Figure 2:**
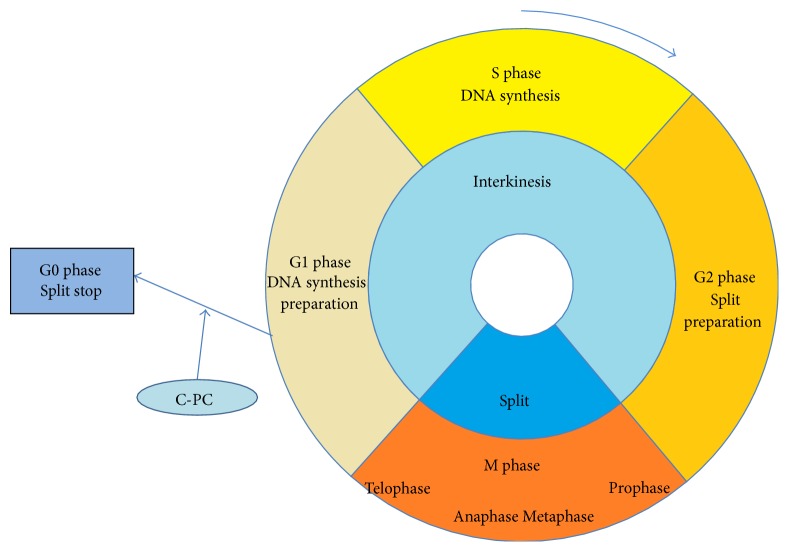
Mechanism of C-PC-mediated cell cycle disruption.

Thangam et al. [45] found through fluorescence and phase contrast microscopy that C-PC displays typical apoptotic characteristics, such as DNA fragmentation, nuclear condensation, membrane blebbing, and cell shrinkage. The application of C-PC in human tumor cell can arrest cell cycle at the G0/G1 phase and block DNA synthesis, indicating inhibition of tumor cell proliferation. Basha et al. found the same results [46]. Moreover, Yong et al. [47] studied the antitumor activity of C-PC against HeLa cells and found that the inhibition rate reaches up to 31% at a concentration of 80 mg/L; they preliminarily concluded that the inhibitory mechanism changes the S or M phase into G1 phase, thereby attenuating DNA synthesis, that is, tumor cell proliferation inhibited by suppressing DNA proliferation.  Figure 2 illustrates the mechanism of C-PC-mediated cell cycle disruption.

#### 4.1.2. Regulating Related Genes and Protein Expression

**Figure 3:**
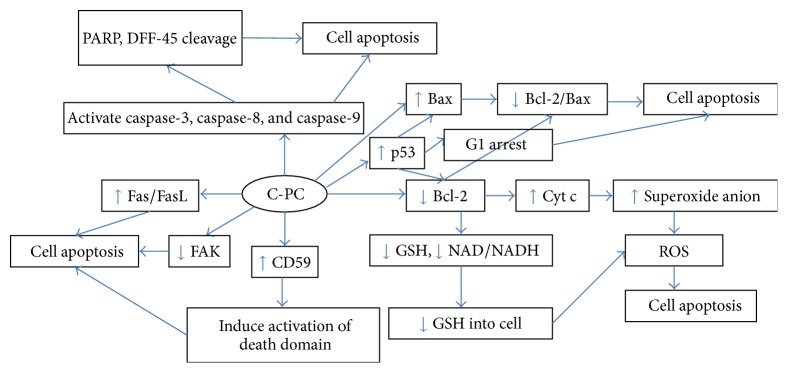
Regulation of C-PC on some genes and proteins and their relationship.

One important cause of cancer is that the inhibition of cell apoptosis is programmed by gene regulation. Numerous reports have revealed that several genes, such as antioncogene p53, proapoptotic gene Fas/FasL, nuclear transcription factors, Bcl-2, and caspase families, can promote or inhibit apoptosis [48, 49].  Table 2 lists some of the common proapoptotic and antiapoptotic genes. Pardhasaradhi et al. found that the Bcl-2 transcription mediated by C-PC in AK-5 cells inhibits cell apoptosis [50]. Overexpression of Bcl-2 can inhibit the production of reactive oxygen species (ROS). Therefore, C-PC-induced apoptosis was regulated by Bcl-2 expression through regulating the generation of free radical. Li et al. [51–53] concluded that C-PC can promote the expression of Fas, cell adhesion molecule-1 (ICAM-1, also called CD54) in HeLa cells, and signal transduction of cell apoptosis and control tumor progression and metastasis. Liu et al. [54] studied the antitumor effect of C-PC on Hepal-6 and found that cell apoptosis is induced by downregulating the Fas/FasL ratio. Cytochrome c (Cyt c) is a component of electron transport in biological oxidation. The study showed that Cyt c released from the mitochondria is related to apoptosis and can induce cell apoptosis. Poly-ADP-ribose polymerase (PARP) is a DNA repair enzyme that plays an important role in DNA damage repair and cell apoptosis; it is also considered an important indicator of apoptosis. Reddy et al. [55] studied the effect of C-PC on RAW264.7 induced by lipopolysaccharide (LPS) and indicated that C-PC can cleave PARP and promote Cyt c release from the mitochondria into the cytoplasm. Another scholar investigated the effect of C-PC on K562 cells and found that the apoptosis of tumor cells is mediated by Cyt c release and PARP cleavage [56]. Caspase, an aspartic acid protease containing cysteine, is responsible for the selective cutting of certain proteins, causing cell apoptosis. Ying et al. [57] investigated the induction of C-PC during the apoptosis of HEP-2 cells and found that caspase-3, caspase-8, and caspase-9 are activated; in addition, the mRNA levels of Bax, Fas, and p53 are upregulated after treatment with C-PC, thereby promoting the signal transduction of apoptosis and eventually apoptosis. Li et al. [58] found that C-PC can inhibit SKOV-3 cell proliferation in a time- and dose-dependent manner. A proteomics research identified 15 differentially expressed proteins between the treatment and control group, but the specific target proteins must be further investigated. CD59 is a complement regulatory protein that is associated with the occurrence of trauma, immune disorder, and tumor. C-PC exerts dose-dependent antitumor effects on HeLa cells containing the CD59 gene [51–53]. C-PC could promote the expression of the CD59 protein, induce the activation of the death domain, and suppress tumor cell proliferation. The regulatory effects of C-PC on some genes and proteins and their relationship with each other are systematically shown in Figure 3.

#### 4.1.3. As a Selective Cyclooxygenase-2 (COX-2) Inhibitor

COX-2 is an induced enzyme that is highly expressed in inflammation and tumor cells [59, 60]. Recent studies have found that COX-2 is closely associated with tumor formation [61] and progression [62–65], as well as tumor angiogenesis [66–68] and metastasis [62]; thus, COX-2 inhibitors are possibly a new target for tumor therapy. COX-2 inhibitors are speculated to demonstrate two anticancer mechanisms. The anticancer activity of COX-2 inhibitors is previously known to be dependent on blocking COX-2 pathway by reducing the formation of COX-2 products, such as prostaglandins E2 (PGE2) [69, 70]. With the progress in this field of study, numerous COX-2 nondependent pathways have been found. In this work, some targets are presented, including (a) reducing the expression level of the Bcl-2 gene [62], increasing the expression level of the transforming growth factor *β*2 receptor that mediates apoptosis, and enhancing the activity of the E calcium protein that mediates cell apoptosis to reduce tumor cell invasiveness [71]; (b) downregulation of vascular endothelial growth factor (VEGF), thereby inhibiting tumor angiogenesis [72, 73]; (c) reducing the expression of matrix metalloprotease (MMP) and urokinase-type plasminogen activator in tumor cells and its invasion ability to normal tissue [67]. Through the above pathways, COX-2 inhibitors can inhibit cell cycle progression, induce cell apoptosis, and inhibit angiogenesis and metastasis. Studies have also revealed the inhibitory effects of C-PC on 12-O-tetradecanoyl-phorbol-13-acetate induced ODX, COX-2, and IL-6 alteration and explored the role of C-PC in tumor development, promotion, and progression [74]. C-PC as COX-2 inhibitor can dock with VEGF1 and inhibit colon cancer through the angiogenic pathway [75]. Reddy et al. [55] found that the C-PC-induced inhibition of COX-2 can reduce PGE2 level of LPS-stimulated RAW264.7 macrophages; they also reported that C-PC is a more potent inhibitor of COX-2 than celecoxib and rofecoxib. C-PC can downregulate the mRNA expression levels of COX-2 genes in the cochlea and inferior colliculus of mice [76]. Chen et al. obtained similar results [77]. A recent study [78] has revealed that C-PC can reduce PGE2 level. PGE2 reduction can decrease the concentration of intracellular adenosine monophosphate [79] and increase the expression of E-cadherin to reduce the occurrence of tumor malignant behavior. Moreover, PGE2 reduction can promote the proliferation of T and B immune cells to improve immunity [80].

### 4.2. Antitumor Effects of Peptides and Subunits of C-PC

Although C-PC demonstrates an active antitumor effect, its heavy molecular weight and complex secondary structure hinder precise determination of small antitumor molecules and their mechanism. Thus, many scholars attempted to obtain different enzymatic hydrolysate and subunits by further processing C-PC. Wang et al. [81] isolated C-PC peptide through enzymatic hydrolysis by column chromatography to obtain four groups of peptide components, and they determined the effects of C-PC, enzymatic hydrolysis, and the four isolated groups of peptide components on HeLa and 293T tumor cells. They found that the different peptide groups demonstrate varying inhibitory effects on cancer cells; groups 1 and 4 showed better tumor inhibitory effect on HeLa cells than the two other groups, whereas the best tumor inhibitory effect on 293T was demonstrated by group 4. However, this study only performed preliminary enzymatic hydrolysis and obtained few groups of unknown structures and then determined the effect of each group on the tumor cells. The specific components of these groups, as well as the structure and the mechanism of tumor suppression, must be further studied. Zhang et al. [82, 83] obtained C-PC subunit through a series of separation methods and then observed the influence of C-PC and *α* and *β* subunits of C-PC on the growth of the lung cancer cell line SPC-A-1. The results showed that the *β* subunit demonstrates a better effect compared with the *α* subunit. Subhashini et al. [56] studied the proliferation inhibition and apoptosis induction of integrated *β* subunit in different tumor cells and found that the *β* subunit can react to membrane binding tubulin and glyceraldehyde-3-phosphate dehydrogenase (GAPDH) to activate caspase-3 and caspase-9. The reduction of GAPDH can prevent the entry into S phase of the cell cycle and arrest cell cycle in the G0/G1 phase, thereby inhibiting tumor cell proliferation.

### 4.3. Use of C-PC Combined with Other Drugs

#### 4.3.1. Combination with Piroxicam

Piroxicam is a traditional nonsteroidal anti-inflammatory drug that is used against rheumatism and rheumatoid arthritis. The combination of piroxicam and C-PC on rat colon carcinogenesis induced by 1,2-dimethylhydrazine dihydrochloride reduces the number and size of tumors, increases the tumor inhibition rate compared with single drug treatment, and reduces drug toxicity and side effects; thus, this combination prevents tumor progression [75, 84–89].

#### 4.3.2. Combination with All-Trans Retinoic Acid (ATRA)

ATRA is often used to treat skin diseases and can also induce tumor cell differentiation and apoptosis. Li et al. [90, 91] investigated the in vitro and in vivo antitumor effect of the combination of C-PC and ATRA on A549 lung cancer cells. Their results showed that such a combination is better than that of single drug treatment, the dosage of ATRA is greatly reduced, and there were no obvious toxic side effects.

#### 4.3.3. Combination with Topotecan (TPT)

TPT is a topoisomerase I inhibitor that can be used to treat nearly all solid tumors. Investigation on the effect of C-PC and TPT on prostate cancer showed that the effect of 10% TPT combined with C-PC is considerably greater than that of normal dosage TPT; this finding is attributed to the increase in ROS and caspase-3/caspase-9 expression [92], showing that this combination offers favorable antitumor application prospects.

#### 4.3.4. Combination with Doxorubicin (DOX)

DOX is an antitumor antibiotic that inhibits DNA and RNA synthesis and demonstrates a wide range of applications. Nishanth et al. [93] and Roy et al. [94] studied the effects of DOX combined with C-PC on the hepatocellular carcinoma cell line HepG2. The results show that the combination displays better antitumor effect and less toxic side effects than single drug treatment. These findings demonstrate that the combination of C-PC and other anticancer drugs exhibits obvious advantages over single drug treatment, although these combinations must be further investigated.

### 4.4. Application in PDT

PDT is a method that produces ROS via selective uptake and retention of photosensitive substance in malignant tumor tissues of organisms and via photooxidation of biological molecules, resulting in the death of tumor cells. C-PC is a photosensitive material that can produce singlet oxygen and other oxygen free radicals under excitation of suitable light wavelength [95]. Morcos et al. first proposed the use of C-PC in the PDT tumor [96] and confirmed the C-PC subunit to demonstrate photosensitive effects [97, 98]. Wang et al. [99] explored the effect of C-PC-mediated PDT on the human hepatocellular carcinoma cell line SMMC-7721 and found that tumor inhibition rate is the highest when C-PC and laser are used simultaneously. Li et al. [100, 101] investigated the role of C-PC in PDT of mouse HeLa cell tumor and breast cancer MCF-7 cells and showed that this mechanism induces tumor cell death by simultaneously enhancing the immune system and initiating the apoptotic signal transduction pathway in the cell.

## 5. Antioxidation

Romay first reported the antioxidant and anti-inflammatory properties of C-PC [102] and showed that C-PC can effectively eliminate hydroxyl free radicals and oxygen free radicals. This finding was also proven by Paloma et al. [103]. Free radicals are involved in the occurrence of many diseases, including inflammation, atherosclerosis, cancer, reperfusion injury, and other disorders caused by oxidative stress [104–106].

### 5.1. Neuroprotective Effect

Reduction in antioxidant capacity and increase in reactive oxygen free radicals are largely associated with the aging of human organs and neurodegenerative diseases [107–110]. Some animal models injected with superoxide dismutase (SOD) showed that SOD can inhibit inflammatory response. In addition, SOD can increase the immune function of some molecules in vitro in immune cells of animal as well as human body [111]. Many clinical trials have reported that cytokine expression is significantly increased in the cerebrospinal fluid and brain tissue of patients with brain injury or infarction [105, 112]. C-PC can decrease infarct size and increases behavior disorder in rats with cerebral artery obstruction [113]. This study established 2D and 3D astrocyte tissue models to determine the effect of C-PC on upregulation of antioxidant enzymes (e.g., SOD, catalase (CAT), brain-derived neurotrophic factor, and brain-derived neurotrophic factor), relief of inflammation factors (e.g., IL-6, IL-1*β*, and glial scar), and improvement of 3D neurons activity. Moreover, C-PC can improve the survival and proliferation ability, weaken the apoptosis of oxidized astrocytes and free radical scavenging ability, and cause no damage to the normal astrocytes and neurons. Mitra et al. [114] compared the protective effects of C-PC and N-acetylcysteine (NAC, a neuroprotective drug) on tributyltin chloride-induced neurotoxicity. They found that both of them can reduce oxidative stress and inflammation, although their mechanisms vary; NAC can effectively regulate enzymes related to the oxidation pathway, whereas C-PC resists ROS. Marín-Prida et al. analyzed the effect of C-PC on Ca^2+^/phosphate induced rat brain mitochondrial damage and showed that C-PC prevents the dissipation of membrane potential, increases ROS levels, and releases proapoptotic Cyt c. Another study revealed that C-PC exerts antioxidant activity by maintaining the activities of cellular antioxidant enzymes, including total glutathione peroxidase (GPx) and GPx-Se, and by increasing reduced glutathione in cells against iron-induced oxidative stress [115]. Therefore, C-PC is a potential neuroprotective agent that can be applied to treat oxidative stress-induced neuronal injury in neurodegenerative diseases, such as ischemic stroke, Alzheimer's disease, and Parkinson's disease [116, 117].

### 5.2. Hepatoprotective Effect

 Vadiraja et al. [118] investigated the pharmacological activities of C-PC on rat liver toxicity induced by R-(+)-pulegone and carbon tetrachloride and showed that C-PC can significantly reduce the liver toxicity caused by a large number of free radicals. Loss of microsomal cytochrome P450, glucose-6-phosphatase, and aminopyrine-N-demethylase was significantly reduced, suggesting that C-PC protects the liver enzymes. Further studies have shown that C-PC can obviously reduce the peroxidation of tryptophan and lipid and improve brain CAT and GPx activities. This finding indicates that C-PC can reduce hepatic brain injury induced by thioacetamide through increased antioxidant activity [119]. A recent study has evaluated the effects of C-PC on Kupffer cell function and showed that C-PC can significantly reduce phagocytosis and the associated respiratory burst activity that may contribute to the abolition of the response of oxidative stress-induced tumor necrosis factor alpha-*α* (TNF-*α*) and nitric oxide (NO) production induced by hyperthyroid state [120]. Another study has revealed that the hepatoprotective mechanism of C-PC is related to the blockage of inflammatory infiltration by inhibiting the expression of tumor growth factor-beta 1 and hepatocyte growth factor [121].

### 5.3. Renoprotective Effect

C-PC can inhibit cisplatin-induced renal toxicity and oxidative stress in a dose-dependent manner, and its protective effect is associated with the attenuation of oxidative stress and the preservation of the activities of antioxidant enzymes [122]. C-PC can inhibit the activities of antioxidant enzymes, GPx, glutathione reductase, glutathione-S-transferase, and CAT in the kidney. In addition, C-PC is a scavenging agent of a series of active substances. Another study reported the same results and also revealed that the mechanism involves, at least in part, the suppression of phosphorylated extracellular signal regulated kinase, Bax, caspase-9, and caspase-3 [123]. Farooq et al. [124] found that C-PC can prevent cellular damage induced by oxalic acid-mediated oxidative stress in canine kidney cells and decrease ROS and lipid peroxidation in cells. C-PC provides significant protection from mitochondrial membrane permeability and increases ATP production. Moreover, C-PC can prevent the occurrence of diabetic nephropathy by inhibiting NADPH dependent superoxide production in cultured renal mesangial cells [125].

### 5.4. Cardiovascular Protective Effect

Lipid metabolism, oxidative stress, and mitochondrial damage play an important role in cardiovascular disease (CVD). Riss et al. confirmed that C-PC effectively improves inflammatory damage caused by oxidative stress in atherosclerotic animals by inhibiting the activity of free radicals and the formation of COX-2 to increase the levels of antioxidant enzymes in the body and by regulating blood lipid [126]. Sheu et al. [127] investigated the antioxidant effect and lipid metabolism of C-PC and found that C-PC effectively lowers serum cholesterol, total cholesterol, triglyceride, low-density lipoprotein, glutamate-oxaloacetate transaminase, and glutamate-pyruvate transaminase. In addition, C-PC was found to increase the activities of CAT, SOD, and GPx. The lipid-lowering and antioxidant effects of C-PC suggest its roles in CVD prevention and atherosclerotic formation. Li [128] found that C-PC can inhibit the progress of atherosclerosis, and the antiatherosclerotic effects of C-PC might be enhanced by promoting CD59 expression, preventing smooth muscle cell proliferation and endothelial cell apoptosis, reducing blood fat levels, and inhibiting the development of atherosclerosis.

### 5.5. Elimination of Cataract

Age related cataract is the leading cause of blindness associated with the accumulation of oxidative stress in the eye lens. A study on the regulatory effect of C-PC on sodium selenite-induced cataract in rats revealed that C-PC can adjust the in vivo and in vitro antioxidant enzyme levels, thereby reducing oxidative stress and the incidence of sodium selenite-induced cataract [129]. Another scholar observed secondary changes in electrolyte levels, mean activities of antioxidant enzymes (i.e., SOD, CAT), and reduced glutathione during sodium selenite-mediated cataractogenesis in rats; this scholar also found that the deleterious effects of sodium selenite toxicity can be restored by simultaneous treatment with C-PC [130]. Further study found that the mechanism involves transcriptional regulation of the lens crystallin, redox genes, and apoptotic cascade mRNA expression, thereby maintaining lens transparency [131]. In addition, apoptosis of lens epithelial cell (LEC) plays an important role in cataract formation. Qu et al. investigated the protective effects of C-PC on human LEC and suggested that C-PC suppresses D-galactose-induced human LEC apoptosis through the mitochondrial pathway, which involves p53 and Bcl-2 family protein expression, and the protein response pathway, which involves glucose regulated protein 78 and chop protein expression [132].

## 6. Anti-Inflammation

C-PC, being a selective COX-2 inhibitor, displays certain hepatoprotective, anti-inflammatory, and antiarthritic properties [133]. The anti-inflammatory effect of* S. platensis* was first reported by Remirez et al. [134]. Romay et al. [135, 136] have recently reported the anti-inflammatory and scavenging oxygen free radicals effects of C-PC. A report has shown that the anti-inflammatory effect of C-PC was dosage-dependent and that it can reduce the inflammatory tissue edema in 12 types of inflammatory cells in an experimental model. Nutrition preparation containing C-PC treatment to osteoarthritis was studied and compared with the anti-inflammatory drug carprofen. Results indicated that this preparation can reduce various inflammatory cytokines, such as TNF-*α*, interleukin-6 (IL-6), MMP-3, NO, and sulfated glycosaminoglycans [137]. All of these inflammatory cytokines are closely associated with the occurrence and development of inflammation. Researchers studied the effect of C-PC on LPS-induced microglia and found that the mRNA expression levels of inducible NO synthase (iNOS), COX-2, TNF-*α*, and IL-6 are downregulated and that the release of lactate dehydrogenase significantly decreases [77]. Thus, C-PC can inhibit the expression of inflammation-related genes in LPS-stimulated BV-2 microglial cells. Shih et al. [138] discovered that C-PC can inhibit overexpression of NO and PGE2 by downregulating the expression of iNOS and COX-2 and reducing the formation of TNF-*α* and the infiltration of neutrophils into inflammation sites. This result indicates that C-PC displays an anti-inflammatory potential, which was also proven by another scholar [139]. Another study revealed the relationship between inflammation activity and oxygen free radical scavenging [140]. In this study, reduced myeloperoxidase and no toxicity were found in male SD rats with ear edema; thus, C-PC is a potential natural anti-inflammatory agent. Another scholar has speculated that the inhibitory effect of C-PC on allergic inflammatory response is mediated by the inhibition of histamine release from mast cells [141]. This study assessed the inhibitory effects of C-PC on induced allergic inflammatory response and histamine release from isolated rat mast cells and discovered that C-PC significantly reduces histamine release.

## 7. Immunomodulatory Effect

Zhang et al. [5] showed that C-PC can improve erythropoietin activity of cells and then directly stimulate the formation of colony forming unit-erythroid, which will stimulate bone marrow hematopoiesis. Peng et al. [42] showed in their animal experiments that C-PC can enhance the activity of lymphocytes, the immunity of an organism, and the body's ability to prevent and resist disease. Test results have confirmed that C-PC can promote phytohemagglutinin-stimulated lymphocyte transformation, recover the E-rosette forming ability of T cell after damaging by cyclophosphamide, and significantly improve the number of antibody-forming cells and their abilities to produce antibodies in normal rats and immune hypofunction mouse spleen cells treated with hydrocortisone [142]. A study of C-PC-mediated PDT on rat tumor model and in vivo and in vitro apoptosis mechanism of MCF-7 cells showed that C-PC can enhance the proliferation of immune organs and immune cells [101]. This result indicates that C-PC can promote immune function and resist diseases. Another research investigated the effect of C-PC on mucosal and immune system response and allergic inflammation in C3H/HeN and BALB/cA mice; the results suggest that C-PC enhances biological defense activity against infectious diseases by sustaining the functions of the mucosal immune system and reduces allergic inflammation suppressing antigen-specific lgE antibody [143]. In addition, C-PC plays a role in autoimmune disorders [144]. In this study, C-PC was used to treat experimental autoimmune encephalitis (EAE) and the result showed that C-PC can prevent or downgrade EAE expression and upregulate the expression of key markers for regulatory T cell (Treg): fork head protein 3, CD25, IL-10, and TGF-*β*. In addition, C-PC might act as a neuroprotector that reverses damage in neurodegenerative disorders of the central nervous system, thereby improving the myelin and axonal damage of EAE. Thus, C-PC demonstrates a therapeutic potential for multiple sclerosis and may lead to effective therapies by activating Treg.

## 8. Safety of C-PC

C-PC is a natural pigment protein isolated from marine alga. C-PC is nontoxic and noncarcinogenic [145] with LD_50_ of >30 g/kg (rat, per os). In a previous study, natural C-PC was given with dosage of 4.00, 40, and 0.12 g/kg for 12 weeks via gavage to SD rats [146], which exerted no adverse effect on the body weight, diet, and water-drinking situation of rats. Regular detection of blood routine examination revealed that red blood cells, platelets, and white blood cells, as well as blood biochemical indices such as glutamic-pyruvic transaminase, glutamic-oxaloacetic transaminase, alkaline phosphomonoesterase, total bilirubin, and serum creatinine, are within the normal range and are not dose-dependent with intragastric samples. No obvious swelling, necrosis, or inflammatory reaction can be observed in the liver, spleen, kidney, or any other important organ tissues and main organs. These results indicate that the oral administration of C-PC elicits no liver toxicity. After 4-week recovery, the aforementioned indices showed no obvious abnormalities. In addition, no significant difference was observed between the high, medium, and low dosage groups and the control group treated with distilled water (*P* > 0.05). This result suggests that the above indicators exert no obvious residual effects and secondary toxicity changes after stopping intragastric administration. Comparison of the results of related literature, along with rat chronic toxicity test results, showed that natural C-PC exerts no oral toxicity and thus is a nontoxic substance, furthering its value to be developed as a functional food and drug.

## 9. Discussion

In this work, we reviewed the separation and purification, physicochemical property, physiological and pharmacological activities, safety, and some applications of C-PC. On the one hand,* S. platensis* contains numerous proteins, which are either soluble or insoluble in water and other water-soluble substances, such as* S. platensis* polysaccharides and vitamins B and C. Thus, the extraction and purification of C-PC are multifarious and are limited in the laboratory, which hamper the improvement of purity and high-volume production. Technology conditions must be further improved. On the other hand, all of these isolation and purification methods are assessed only in terms of the extraction rate and initial degree of C-PC; changes in property or structure are studied which possibly influence various pharmacological effects of C-PC that are not investigated. The molecular weight and amino acid sequence of the *α* and *β* subunits of C-PC have already been studied by many scholars, but authoritative and accurate conclusion has not yet been published. C-PC is a pigment protein, and the activity preservation of C-PC is important. However, whether the activity of C-PC is from spatial structure is rarely investigated. All of those are closely related to the target of C-PC activities on all respects, so complex and thorough research is necessary. In addition, C-PC demonstrates various pharmacological activities, and the mechanisms underlying their effects have been extensively investigated by scholars. C-PC can inhibit COX-2, modulate the expression of some genes or proteins, and scavenge free radicals, which are associated with diseases, such as tumor, inflammation, CVD, and cataract. However, the main mechanism involved in fighting a specific disease and the mechanism of interaction between different diseases have not yet been well explained. Elucidating the interaction among these mechanisms may improve the effective usage of C-PC and avoid some side effects, although no obvious side effects and toxicity have been found to date. Given the pharmacological effects of C-PC and its property of being natural, nontoxic combination of C-PC with other known drugs that treat a particular disease well has become a trend. Whether combined with antitumor or anti-inflammatory drugs, C-PC can enhance therapeutic effects, minimize side effects and toxic reactions, and reduce drug dosage. In fact, drug combination has already been a trend because of its advantages, and the combination of C-PC with a great number of drugs is worth investigating. C-PC being a protein is not very stable, and its LC_50_ is higher when used as an antitumor drug; thus, studies on preparation are also vital either for stability or for absorption. At present, some studies focused on the new preparation of C-PC and its application in the production of new tumor-suppressing materials, such as liposomes, nanoparticles, and carbon nanotubes. However, these studies are limited to simple pharmacological evaluation and are not comprehensive. Experiments on other aspects, such as cell uptake, distribution, and metabolism, are also needed. Cancer is a serious and unconquered threat to human health. Although some cancer treatment has gained some satisfactory results, some unsatisfactory cases, such as solid tumors, still exist. C-PC can significantly promote tumor cell apoptosis and inhibit tumor growth and is safe and nontoxic; thus, C-PC is especially suitable for the prevention and treatment of cancer. In the context of rapid development of new formulations and materials, a new glimmer for cancer treatment has been brought into light. Surgical treatment, radiotherapy, and chemotherapy can eliminate tumor lesion, destroy a certain number of metastatic tumor cells and residual cancer cells, and play positive roles in changing the condition, in which the factors promoting tumor development are greater than the vicious circle of immune function. However, these three treatments cannot eliminate all cancer cells in their division stage, and the elimination of the rest of the tumor cells still depends on the body's normal immune function. The essence of tumor recovery is the recovery of self-healing ability; that is, patients can receive better treatment effects if their immune system can recover as soon as possible after surgery, radiotherapy, and chemotherapy and display a relatively good tumor killing ability (TKA) and certain self-healing ability. The Japanese literature has reported that 90% of cancer patients with TKA index >800 are cured. C-PC strongly promotes immunity and thus can be used for adjunctive tumor treatment for surgery, radiotherapy, and chemotherapy. In conclusion, C-PC offers a great development prospect either as a functional food or as a pharmaceutical product.

## 10. Conclusion

C-PC is deep blue powder obtained from marine algae. C-PC is not only a type of protein, but also an excellent natural edible pigment, food, and cosmetic additive and a health product. C-PC demonstrates a series of physiological and pharmacological activities (e.g., antitumor, antioxidation, anti-inflammation, and immune regulation) without causing toxicity and harm and has been used in some fields; however, a great number of related applied products have not yet been widely studied and applied, especially in the medicinal field. To comprehensively apply C-PC, researchers must further rigorously design and conduct studies.

## Figures and Tables

**Table 1:** Spectral property of C-PC (R-PE referred to R-phycoerythrin).

Pigment	Absorbance maximum (nm)	Fluorescence emission (nm)	Molecular weight (kDa)	Absorptivity (L/g·cm)	Fluorescence absorbance (related to R-PE)
C-PC	615	647	220	7.0	0.15

**Table 2:** Common pro- and antiapoptosis genes.

Bcl-2 family	Bcl-2, Bcl-XL, Bcl-W, Mcl-1, CEd-9, and so forth	Antiapoptosis
	Bax, Bak, Bcl-XS, Bad, Bik, Bid, and so forth	Proapoptosis
Caspase family	Caspase-2, caspase-3, caspase-6, caspase-7, caspase-8, caspase-9, caspase-10, and so forth	
Others	Fas, p53, NPRL2, PKIP, THY1, and so forth	
